# In-Depth Analysis of the Impact of Different Serum-Free Media on the Production of Clinical Grade Dendritic Cells for Cancer Immunotherapy

**DOI:** 10.3389/fimmu.2020.593363

**Published:** 2021-02-05

**Authors:** João Calmeiro, Luís Mendes, Iola F. Duarte, Catarina Leitão, Adriana R. Tavares, Daniel Alexandre Ferreira, Célia Gomes, João Serra, Amílcar Falcão, Maria Teresa Cruz, Mylène A. Carrascal, Bruno Miguel Neves

**Affiliations:** ^1^ Faculty of Pharmacy, University of Coimbra, Coimbra, Portugal; ^2^ Center for Neuroscience and Cell Biology (CNC), University of Coimbra, Coimbra, Portugal; ^3^ CICECO, Aveiro Institute of Materials, Department of Chemistry, University of Aveiro, Aveiro, Portugal; ^4^ Department of Medical Sciences and Institute of Biomedicine (iBiMED), University of Aveiro, Aveiro, Portugal; ^5^ Coimbra Institute for Clinical and Biomedical Research (iCBR), Faculty of Medicine, University of Coimbra, Coimbra, Portugal; ^6^ Center for Innovation in Biomedicine and Biotechnology (CIBB), University of Coimbra, Coimbra, Portugal; ^7^ Tecnimede Group, Sintra, Portugal; ^8^ Coimbra Institute for Biomedical Imaging and Translational Research (CIBIT), University of Coimbra, Coimbra, Portugal

**Keywords:** cancer immunotherapy, dendritic cells vaccines, clinical grade serum-free media, metabolic activity, dendritic cell differentiation, CTL priming, dendritic cell-NK cell crosstalk

## Abstract

Dendritic cell (DC)-based antitumor vaccines have proven to be a safe approach, but often fail to generate robust results between trials. Translation to the clinic has been hindered in part by the lack of standard operation procedures for vaccines production, namely the definition of optimal culture conditions during *ex-vivo* DC differentiation. Here we sought to compare the ability of three clinical grade serum-free media, DendriMACS, AIM-V, and X-VIVO 15, alongside with fetal bovine serum-supplemented Roswell Park Memorial Institute Medium (RPMI), to support the differentiation of monocyte-derived DCs (Mo-DCs). Under these different culture conditions, phenotype, cell metabolomic profiles, response to maturation stimuli, cytokines production, allogenic T cell stimulatory capacity, as well as priming of antigen-specific CD8^+^ T cells and activation of autologous natural killer (NK) cells were analyzed. Immature Mo-DCs differentiated in AIM-V or X-VIVO 15 presented lower levels of CD1c, CD1a, and higher expression of CD11c, when compared to cells obtained with DendriMACS. Upon stimulation, only AIM-V or X-VIVO 15 DCs acquired a full mature phenotype, which supports their enhanced capacity to polarize T helper cell type 1 subset, to prime antigen-specific CD8^+^ T cells and to activate NK cells. CD8^+^ T cells and NK cells resulting from co-culture with AIM-V or X-VIVO 15 DCs also showed superior cytolytic activity. 1H nuclear magnetic resonance-based metabolomic analysis revealed that superior DC immunostimulatory capacities correlate with an enhanced catabolism of amino acids and glucose. Overall, our data highlight the impact of critically defining the culture medium used in the production of DCs for clinical application in cancer immunotherapy. Moreover, the manipulation of metabolic state during differentiation could be envisaged as a strategy to enhance desired cell characteristics.

## Introduction

Dendritic cells (DCs) comprise several subsets of highly specialized professional antigen-presenting cells with superior capacity to acquire, process and present antigens to naïve T-lymphocytes (T-cells) (1). Considering their capacity to elicit strong antitumor immune responses, DCs have been extensively used in immunotherapeutic strategies to fight cancer (2, 3). Currently, there are four main approaches exploring DCs in oncologic treatments: 1) non-targeted protein and nucleic acid-based vaccines captured by DCs *in vivo;* 2) direct targeting of antigens to DCs *in vivo*; 3) vaccines composed of *ex vivo* produced DCs, matured and loaded with tumor antigens; 4) biomaterial based platforms to recruit and program endogenous DCs (4). Among these approaches, *ex vivo* generated DCs are used in nearly 97% of the registered clinical trials, leukapheresis-isolated CD14^+^ monocytic precursors being the primary source to produce monocyte-derived DCs (Mo-DCs) (5).

Notwithstanding the good safety profile of DC antitumor vaccines, the rate of success in inducing clear therapeutic outcomes has been inconstant, with effective responses observed in less than 15% of the cases (5). Several factors have been suggested to explain this outcome: patients with a severely compromised immune system; the multitude of tumor immunosuppressive mechanisms that actively dampen DC functionality; the antigens selected as targets; the limited immunostimulatory abilities of Mo-DCs; and the lack of clinical standard operating procedures (cSOPs) for DCs production (6, 7).

The nonexistence of cSOPs for *ex vivo* manipulation of DCs results in a plethora of protocols that differ in the source of precursors, differentiation and maturation stimuli, antigen nature and loading procedures and, finally, route of administration (5). While extensive research has been performed on the impact of cytokines and growth factors used for DC differentiation and maturation, the relevance of culture media to these processes has been underestimated. Evidence has emerged demonstrating that the metabolism influences DC differentiation, with several connections established between cell metabolic state and their functional specialization [reviewed in (8)]. Hence, it is reasonable to expect that the culture media used during Mo-DCs production may impact their metabolism and inherently their phenotype and functional capacities. Most of our knowledge on *ex vivo* Mo-DC differentiation comes from culture settings comprising fetal bovine serum (FBS). However, for clinical purposes it is crucial to substitute serum or serum components of animal origin, in order to avoid immune reactions and transmission of infectious diseases (9). The use of autologous human serum (HS) can also be undesirable, as many factors that influence DC differentiation and maturation differ between patients and thus contribute to cell product variability (10, 11).

To overcome these limitations, several clinical grade serum-free media (SFM), have become commercially available, allowing to operate according to good manufacturing practice (GMP) conditions. Despite the extensive use of these SFM in the production of clinical grade DCs, very few studies directly compare them for their ability to influence differentiation and cell functional abilities (12–15). In this study, we analyzed the impact of three different SFM (DendriMACS, AIM-V, and X-VIVO 15) on the production of Mo-DCs. We show that the distinct media impact the phenotype, response to maturation stimuli and consequently the immunostimulatory capacities of produced cells. When compared to DendriMACS, immature DCs (iDCs) produced in X-VIVO 15 or AIM-V express higher levels of CD11c, CD86, and major histocompatibility complex (MHC)-I, while presenting lower levels of CD1a and CD1c. Upon stimulation with alpha cytokine cocktail, AIM-V and X-VIVO 15 DCs presented higher expression of CD86, C-C chemokine receptor 7 (CCR7) and superior production of interleukin (IL)-12p70. Such characteristics enable these DCs to polarize CD4^+^ T cells toward T helper cell type 1 (Th1) subset, prime antigen-specific CD8^+^ T cells, and activate natural killer (NK) cells in a superior way. The observed phenotypical and functional differences correlate with the cell’s metabolic status, as revealed by combined metabolomics profiling of culture media and intracellular extracts.

Hence, this study highlights the need of rationally selecting the medium used in the production of clinical grade DCs according to the desired immunostimulatory abilities and therapeutic goals.

## Methods

### Culture Media

Four culture media were tested in this study: AIM-V (Gibco, Waltham, MA USA), X-VIVO 15 (Lonza, Basel, Switzerland), DendriMACS (Miltenyi Biotec, Bergisch Gladbach, Germany) and Roswell Park Memorial Institute Medium (RPMI) 1640 (Gibco, MA, USA). AIM-V, X-VIVO 15 and DendriMACS are SFM, used in clinical DC-based immunotherapy. RPMI is the most used culture medium in pre-clinical DC research and it was supplemented with 10% heat-inactivated fetal bovine serum (FBS), 100 U/ml penicillin, and 100 µg/ml streptomycin, 2 mM glutamax, 1 mM sodium pyruvate, and 1× MEM non-essential amino acids (all from Gibco).

### Cell Isolation and Culture

To obtain human monocytes and T cells, peripheral blood mononuclear cells (PBMCs) were isolated by Ficoll-Paque (GE Healthcare, Chalfont St. Giles, UK) gradient centrifugation from buffy coats of healthy volunteers. Buffy coats were provided by the Portuguese Blood and Transplantation Institute (IPST) following an established protocol allowing access to buffy coats for scientific research with academic purposes only. The buffy coats were not specifically obtained for the present study and were provided without any personal detail from the donor.

Monocytes and T cells were isolated by positive selection using CD14 and CD3 antibody-coated magnetic beads (Miltenyi Biotec), respectively, as described by the manufacturer. T cells were maintained in RPMI medium until co-cultured with DCs. Monocytes were cultured at a density of 1 × 10^6^ cells/ml in the different culture media supplemented with 250 U/ml of IL-4 (Peprotech, London, UK) and 400 U/ml of granulocyte-macrophage colony-stimulating factor (GM-CSF) (Peprotech), for differentiation into immature DCs (iDCs). Each medium was refreshed every 2 days and DCs maturation was induced at day 6 of culture, by adding 50 ng/ml of tumor necrosis factor (TNF)-α (Biolegend), 25 ng/ml of IL-1β (Biolegend), 20 ng/ml of polyinosinic:polycytidylic acid (Poly-I:C) (Novus Biologicals, Abingdon, UK), and 100 ng/ml of interferon (IFN)-γ (Peprotech).

### Flow Cytometry

Cell staining was performed using fluorescence-conjugated antibodies, specifically CD1a-Alexa Fluor 488, CD14-PE, CD11c-APC, CD1c-FITC, CD16-APC, CD86-Alexa Fluor 488, CD83-PE, CD80-PerCP/Cy5.5, CD40-APC, human leucocyte antigen (HLA)-DR-Alexa Fluor 488, HLA-ABC-APC, CCR C-C chemokine receptor 1 (CCR1)-Alexa Fluor 488, CCR2-PerCP/Cy5.5, CCR5-APC, CCR7-PerCP/Cy5.5, chemokine receptor (CXCR4)-PE, CD3-PE, CD4-PerCP/Cy5.5, CD8-APC, CD69-FITC, CD25-APC, forkhead-box-P3 (FoxP3)-FITC, T-box protein expressed in T-cells (T-bet)-PE (all from Biolegend). Isotype-matched antibodies were used as controls. Briefly, 0.2 × 10^6^ monocytes, DCs or T cells were washed and stained with 3 µl of fluorescence-conjugated antibodies in phosphate-buffered saline (PBS) + 1% FBS for 30 min at 4°C, in the dark. Cells were subsequently washed, resuspended in PBS + 1% FBS and analyzed in an Accuri C6 flow cytometer (BD Bioscience, San Jose, CA, USA). For intracellular staining, Fix&Perm (Thermo FisherScientific, Waltham, MA, USA), a fixation and cell permeabilization kit, was used as described by the manufacturer. Data were analyzed with FlowJo™ software (version 10) and results presented as percentage of positive cells or mean fluorescence intensity (MFI) after subtraction of isotype control values.

### Phagocytosis Assay

The human bladder cancer cell line, UM-UC3 (ATCC, Manassas, VA, USA), was labeled with 0.75 μM carboxyfluorescein succinimidyl ester (CFSE) in PBS, during 20 min at 37°C. Excess dye was quenched by adding culture medium containing 10% FBS and then cell-washed twice with PBS. Cell death was induced by heating cells at 60°C for 30 min and then apoptotic/necrotic cells were added for 2 h at 37°C, in a 2:1 ratio to iDCs differentiated with the different media. After co-culture, iDCs were labeled with APC-conjugated anti-HLA-DR antibody and their phagocytic capacity was assessed by flow cytometry as the percentage of HLA-DR^+^ DCs positive for CFSE fluorescence.

### Cytokine Analysis

The transcriptional levels of *IL12B*, *TNF, IL10*, and transforming growth factor beta 1 (*TGFB1)* genes were analyzed by quantitative real-time polymerase chain reaction (qPCR) on DCs differentiated and maturated in the different media. RNA was extracted with NZY Total RNA Isolation kit (Nzytech, Lisbon, Portugal), according to the manufacturer’s instructions. RNA concentration was measured by OD260 using a NanoDrop spectrophotometer (ThermoScientific, Wilmington, DE, USA) and samples were kept in RNA Storage Solution (Ambion, Foster City, CA, USA) at −80 °C until further use. cDNA was obtained by reverse transcription of 1 µg of total RNA using the NZY First-Strand cDNA Synthesis Kit (Nzytech). qPCR reactions were performed on a Bio-Rad CFX Connect equipment (Bio-rad, Hercules, CA, USA) and gene transcription changes were analyzed using the built-in CFX Maestro software, as previously described (16). Results were normalized using glyceraldehyde 3-phosphate dehydrogenase (*GAPDH*) as a reference gene, experimentally determined with Genex software (MultiD Analyses AB, Göteberg, Sweden) as the most stable for the used treatment conditions. Primer sequences (Supplementary Table 1) were designed using Beacon Designer software version 7.7 Premier Biosoft International (Palo Alto, CA, USA) and thoroughly tested.

The secretion of IL12p70 by mature DCs and IFN*γ* by T cells after co-culture with matured DCs was analyzed by ELISA Max Deluxe Kits (Biolegend, London, UK), according to the manufacturer instructions.

### Mixed Leukocyte Reaction (MLR)

To assess T cell proliferation, allogenic T cells were stained with CFSE before being co-cultured with matured DCs for 5 days at 10:1 ratio. All co-cultures were carried out in U-bottomed 96-well plates with a final volume of 200 µl of RPMI medium. The percentage of positive T cell subtypes and their activation and proliferation was analyzed by flow cytometry.

At the end of the co-culture period, cells were stained with anti-CD4 and anti-CD8 antibodies. Additionally, anti-T-bet, anti-Foxp3, and anti-CD25 were also used to evaluate Th1 and T regulatory lymphocytes (Treg) subsets. To measure T cell activation, allogenic T cells were co-cultured with matured DCs for 18 h at a 10:1 ratio and then stained for CD4, CD8 and CD69 activation marker.

### Cytolytic Assays

To address the capacity of DCs to prime and expand antigen-specific CD8+ T cells, autologous HLA-A2^+^ CD8^+^ and CD4^+^ T cells were co-cultured for fourteen days at 10:1:1 ratio with matured DCs previously loaded with short (ELAGIGILTV) or long (GHGHSYTTAEELAGIGILTVILGVL) Melan-A peptides. All co-cultures were carried out in 24-well plates with a final volume of 1 ml of X-VIVO 15 supplemented with 10% FBS. The percentage of Melan-A-specific CD8^+^ T cells was analyzed by flow cytometry, using Pro5 HLA-A*02:01 – ELAGIGILTV Pentamer – Biotin (Proimmune, Oxford, UK) as described by the manufacturer. The cytolytic activity was evaluated after 24 h of co-culture of these stimulated T cells and Melan-A-loaded H1650 lung cancer cell line at 50:1 ratio by lactate dehydrogenase (LDH) release assay (Biolegend), as described by the manufacturer.

To assess the effect of each medium in the capacity of DCs to activate NK cells, autologous HLA-A2^+^ NK cells were co-cultured with matured DCs for 24 h at 5:1 ratio. These co-cultures were carried out in U-bottomed 96-well plates with a final volume of 200 µl of RPMI medium. DC-NK cell bidirectional crosstalk was addressed by analyzing the impact of cell interaction on the DC expression levels of CD86, MHC-I and MHC-II and on the NK cell expression levels of CD69 and CD25. The NK cytolytic activity was evaluated after 24 h of co-culture of these stimulated NK cells and H1650 lung cancer cell line or Panc1 pancreatic cancer cell line at 10:1 ratio by LDH release assay.

### Metabolomic Analysis

At the end of the DC differentiation period, medium was collected and centrifuged at 300*g* for 5 min to collect floating cells. Supernatants were stored at −80°C for further extraction and cells were washed 3 times with cold PBS and immediately extracted using a biphasic extraction protocol with methanol/chloroform/water (1:1:0.7), as previously described (17). The resulting polar extracts were dried under vacuum in a SpeedVac concentrator (Eppendorf, Hamburg, Germany) and stored at −80°C until analysis. Cell conditioned media was thawed and subjected to a protein-precipitation procedure as described elsewhere (18). The resulting supernatants were vacuum dried and stored at −80°C until nuclear magnetic resonance (NMR) acquisition. Respective cell-free medium was processed in the same conditions and used to assess metabolite consumption and excretion by cells.

For NMR analysis, the dried samples were resuspended in 600 μl of deuterated PBS (100 mM, pH 7.4) containing 0.1 mM 3-(trimethylsilyl) propanoic acid (TSP-*d_4_*), and 550 μl of each sample were transferred to 5 mm NMR tubes. All samples were analyzed in a Bruker Avance III HD 500 NMR spectrometer (University of Aveiro, Portuguese NMR Network), operating at 500.13 MHz for ^1^H observation, at 298 K, using a 5 mm TXI probe. Standard 1D ^1^H spectra with water presaturation (pulse program “noesypr1d” from Bruker library) were acquired and processed as previously described (19). Metabolite assignment was based on matching spectral information to reference spectra available in Chenomx (Edmonton, Canada), BBIOREFCODE-2–0–0 (Bruker Biospin, Rheinstetten, Germany) and the Human Metabolome Database (HMDB) (20).

Principal Component Analysis (PCA) was carried out in SIMCA-P 11.5 (Umetrics, Umeå, Sweden) to identify sample clusters, while signal integration, performed in Amix-Viewer 3.9.15 (Bruker Biospin, Rheinstetten, Germany), was employed to provide quantitative information on metabolite levels and variations. For media samples, only statistically significant variations (p < 0.05) with an absolute effect size larger than 1 (calculated as described in ref (21)) were considered.

### Statistical Analysis

Results are presented as mean ± standard error of the mean (SEM) of the indicated number of experiments. Comparisons between two groups were made by the two-sided unpaired Student’s t test and multiple group comparisons by one-way ANOVA analysis, with a Tukey multiple comparison post-test. Statistical analysis was performed using GraphPad Prism, version 6 (GraphPad Software, San Diego, CA, USA). Significance levels are as follows: *p <0.05, **p <0.01, ***p <0.001, ****p <0.0001

## Results

### Different SFM Distinctly Impact the Phenotype of iDCs

**Figure 1:**
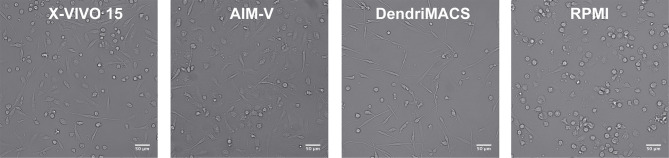
Representative images of immature dendritic cells (iDCs) at day 6 of culture. Monocytes were cultured with GM-CSF and IL-4 in three different serum-free media (SFM)—AIM-V, X-VIVO-15, and DendriMACS—and with FBS-supplemented RPMI, for 6 days. Human iDCs differentiated in SFM are adherent and present a similar elongated morphology, while DCs differentiated in RPMI present a round shape with decreased cell adherence. X-VIVO 15, AIM-V, DendriMACS, RPMI. Images were obtained by wild-field microscope Zeiss AXIO Observer. Magnification: 100×. Scale = 50 μm.

**Figure 2:**
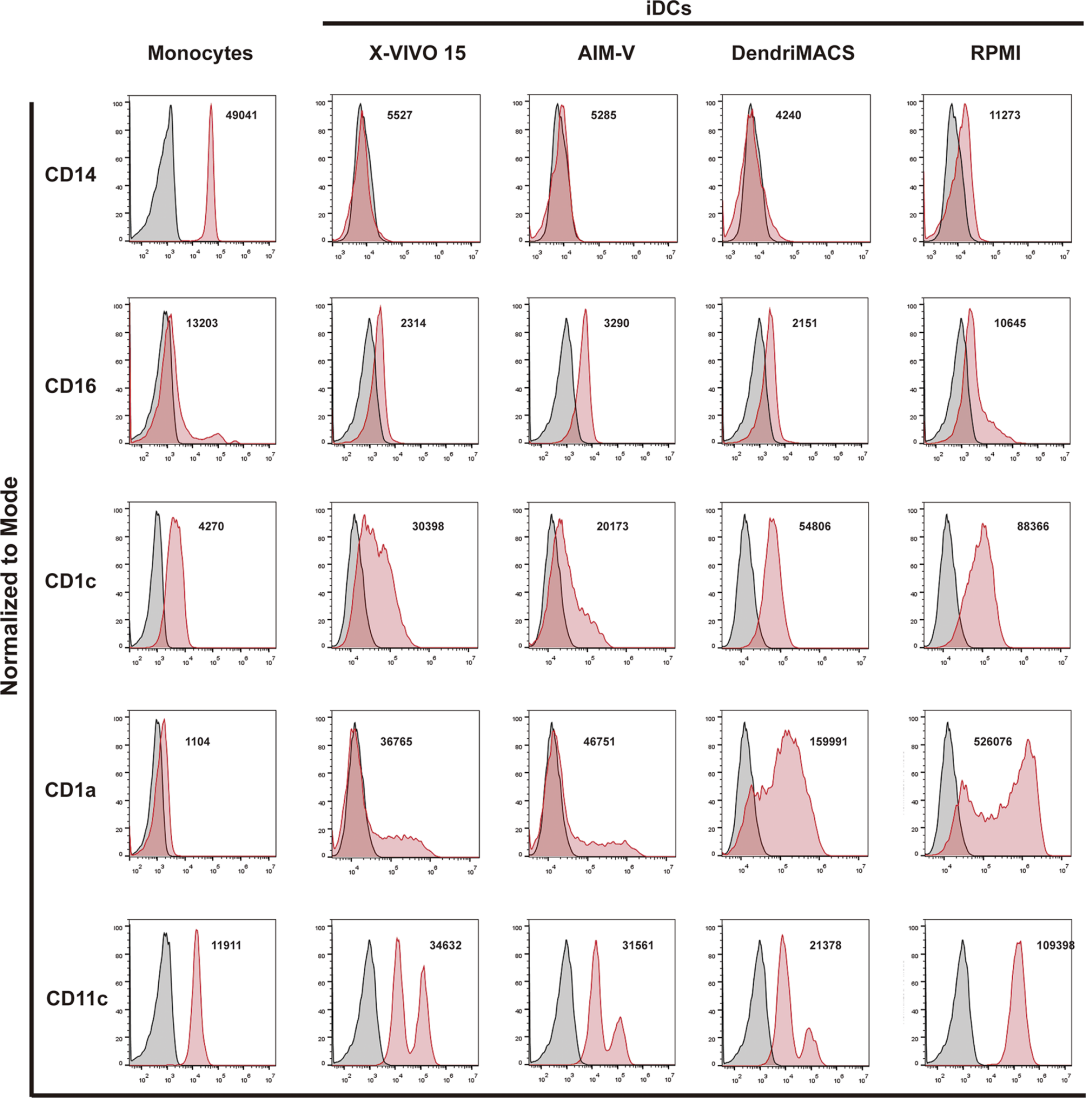
Phenotypic comparison of immature dendritic cells (iDCs) generated in different culture media. Monocytes were cultured with GM-CSF and IL-4 in three different good manufacturing practice (GMP) serum-free media (SFM)—AIM-V, X-VIVO-15, and DendriMACS—and with FBS-supplemented RPMI. After 6 days of culture, expression levels of CD14, CD16, CD1c, CD1a, and CD11c were analyzed by flow cytometry. Viable cells were gated according to their morphological properties. Black line corresponds to isotype control and red line represents specific expression of tested cells. The histograms are representative of at least three independent experiments and the mean fluorescence intensity (MFI) of the performed experiments is presented.

Monocytes were cultured with GM-CSF and IL-4 in three different SFM, specifically AIM-V, X-VIVO-15, and DendriMACS, or with FBS-supplemented RPMI. After 6 days of differentiation, iDCs were analyzed for their morphological appearance and expression levels of CD14, CD16, CD1a, CD1c, and CD11c. The yield of differentiation as well as the viability (>80%) was similar between all four tested media (data not shown). Cells cultured in X-VIVO 15, AIM-V, or DendriMACS were found to be adherent and with elongated shape, in contrast to iDCs differentiated in RPMI that were rounder and mainly non- or loosely adherent (**Figure 1**). Differentiation of monocytes toward an iDC phenotype was observed for all the tested media, as proved by the downregulation of CD14 and CD16 and the increased expression of the DC markers CD1a, CD1c, and CD11c (**Figure 2**). Graphics for mean fluorescence intensity (MFI) as well as statistical analysis are provided in **Figure S1**. However, the expression levels of DC markers are quantitatively different between media. Cells cultured in RPMI present a significant higher expression of CD1a, CD1c, and CD11c, when compared to SFM-iDCs (**Figure S1**). Of all SFM, DendriMACS elicited the highest levels of CD1c and CD1a, while X-VIVO 15 and AIM-V cultured cells presented an increased expression of CD11c. Importantly, all the three SFM induced two distinct populations regarding the expression levels of CD11c, with this output being particularly evident for cells differentiated in X-VIVO 15 (**Figure 2**).

The capacity of DCs to endocyte extracellular material, such as apoptotic/necrotic tumor cells, is critical for their role in antitumor immunity and is an indicator of functional quality of *ex-vivo* differentiated DCs. Therefore, we tested the impact of the different GMP SFM and of RPMI on iDCs capacity to endocyte apoptotic-labeled cancer cells, as previously described (22). The results indicate that iDCs produced with the three SFM present a similar capacity to internalize apoptotic/necrotic tumor cells. After 2 h of co-culture, 23,5% ±5,6 (AIM-V), 30,6% ±3,5 (X-VIVO 15) and 32,2% ±4,1 (DendriMACS) of iDCs presented engulfed material (**Figure S2**). However, these values are significantly lower (p<0,05; p<0,01) than the ones observed for iDCs produced in serum-supplemented RPMI (89,4%). These results clearly indicate that the absence of serum during DC differentiation negatively modulates their endocytic capacity.

### Culture Media Used during DC Differentiation Affects Their Response to Maturation Stimuli

To address whether culture media used during DC differentiation impacts their response to maturation stimuli, cells obtained with the different media were treated with a maturation cocktail frequently used in clinical trials, comprising TNF-α, IL-1β, poly I:C, and IFN-γ. Cell maturation profile was subsequently assessed by analyzing the expression levels of: CD40, CD80, CD83, CD86, MHC-I, and MHC-II; the chemokine receptors CCR1, CCR2, CCR5, CCR7, and CXCR4; and the transcription levels of *IL12B*, *TNF*, *IL10*, and *TGFB.* The production of IL12p70 was also assessed.

**Figure 3:**
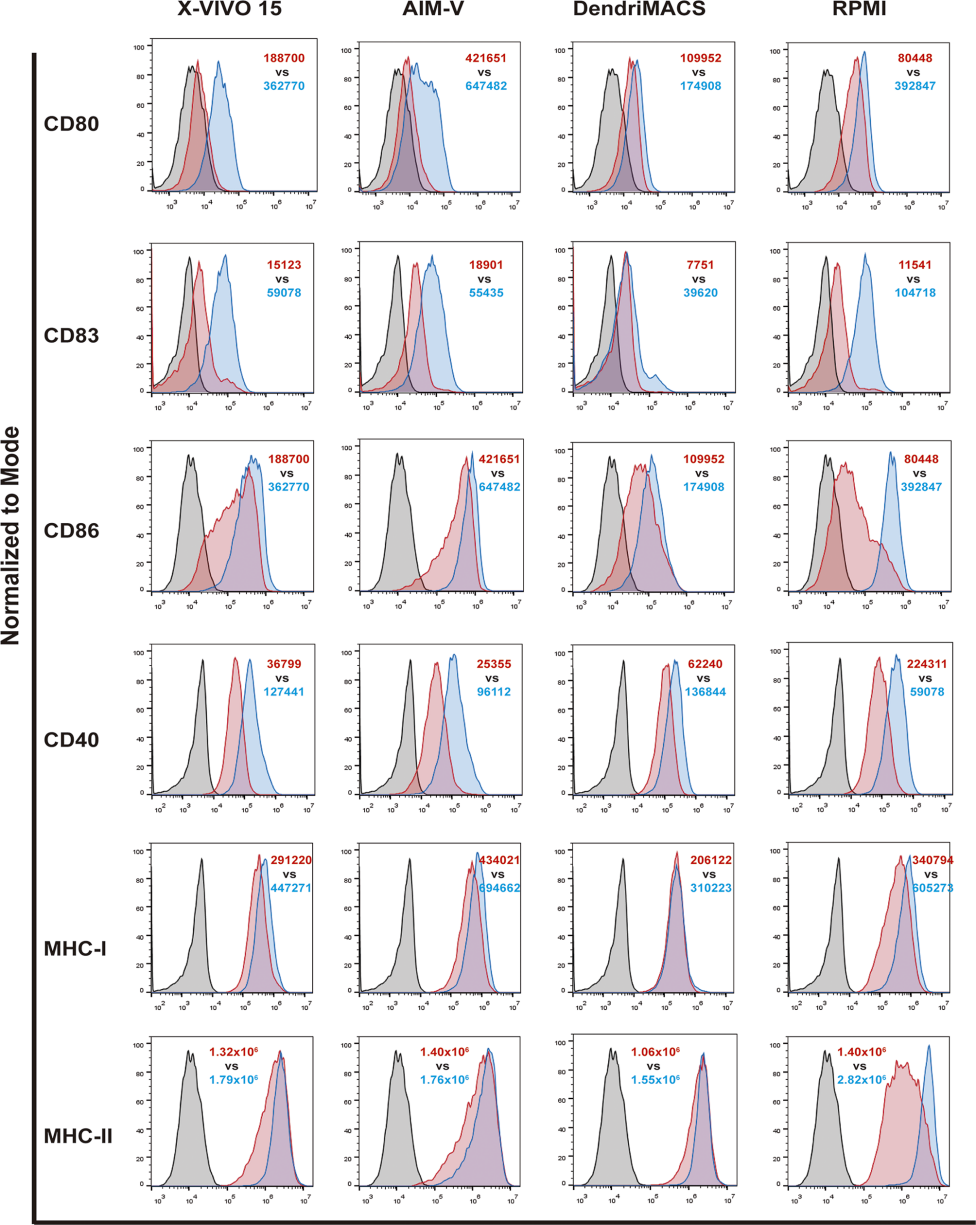
Impact of different culture media on the response of dendritic cells (DCs) to maturation stimuli. DCs differentiated with the four tested media were matured as described in *methods* section. The expression of CD80, CD83, CD86, CD40, MHC-I and MHC-II was assessed by flow cytometry. Black line corresponds to respective isotype control, red line represents specific expression on immature DCs (iDCs) and blue line the expression on mature DCs (mDCs). Final analysis was performed including only viable cells based on their morphological properties. The histograms are representative of at least four independent experiments. In red, MFI values for iDCs and in blue MFI values for mDCs.

Before maturation, the levels of CD83, CD40 and MHC-II were found to be similar between iDCs produced with the four media. In contrast, iDCs differentiated with AIM-V presented a significantly higher basal expression of the co-stimulatory molecule CD86: vs. X-VIVO 15 p<0,001; vs. DendriMACS p<0,0001, and vs. RPMI p<0,0001 (**Figure 3** and **Figure S3**). In a similar way, cells produced in RPMI were shown to express significantly higher levels of CD80 than X-VIVO 15 iDCs (p<0,01) and AIM-V iDCs (p<0,05). Although not statistically different, X-VIVO 15 and AIM-V iDCs consistently presented inferior levels of CD80 and CD40 and a slightly higher expression of MHC-I and CD83.

All the culture media supported an increase in the expression of co-stimulatory and MHC molecules upon treatment of cells with the maturation cocktail. However, the observed increases presented distinct magnitudes, with RPMI-differentiated DCs being the ones that strongly respond to the used stimuli. On the other hand, DCs produced with DendriMACS appear to be somehow resistant to maturation, presenting a smaller increase upon stimulation, particularly for CD86 expression. Cells differentiated with X-VIVO 15 and AIM-V, albeit not maturing as effectively as RPMI DCs, substantially increased the expression of CD40, CD80 and CD83.

**Figure 4:**
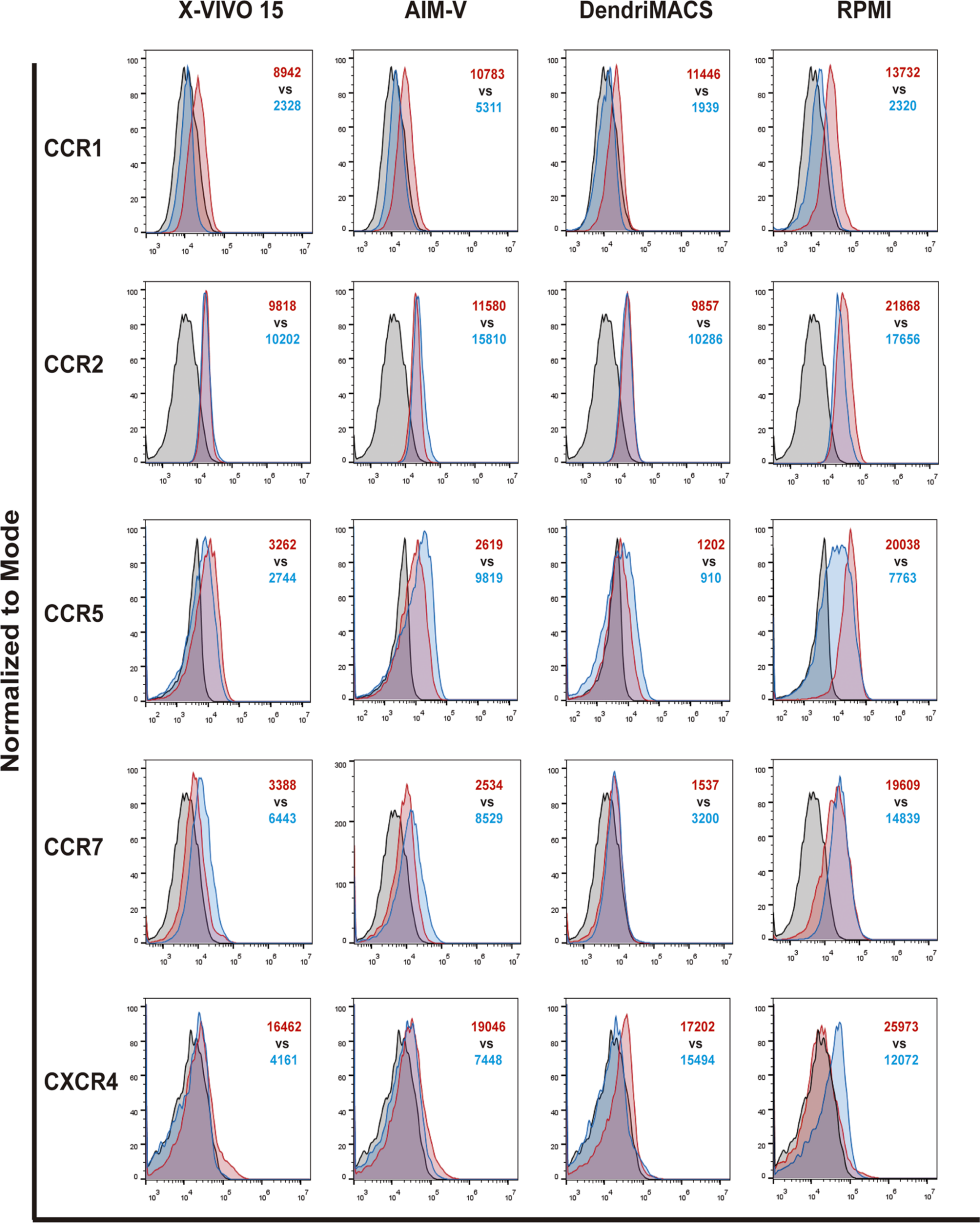
Chemokine receptors profile of immature (iDCs) and mature (mDCs) dendritic cells. CCR1, CCR2, CCR5, CCR7 and CXCR4 expression levels were assessed by flow cytometry. Black line corresponds to respective isotype control, red line represents specific expression in iDCs and blue line the expression on mDCs. Final analysis was performed including only viable cells based on their morphological properties. The histograms are representative of four independent experiments. In red MFI values for iDCs and in blue MFI values for mDCs.

A switch in chemokine receptor profile is another hallmark of DC maturation, allowing their relocation from inflamed tissues to draining lymph nodes (23). To migrate to lymph nodes, maturing DCs should upregulate CCR7 to respond to the lymphoid chemokines C-C motif ligands 19 and 21 (CCL19 and CCL21), while downregulating CCR1, CCR2, and CCR5. Therefore, the impact of culture media on the expression of CCR1, CCR2, CCR5, CCR7, and CXCR4 was evaluated by flow cytometry. As shown in **Figure 4** and **Figure S4**, analyzed media elicited a distinct basal chemokine receptor profile in iDCs and induced different responses upon maturation. Basal levels of CCR2, CCR5 and CCR7 are significantly higher in cells differentiated in FBS-supplemented RPMI, while no differences were observed between iDCs produced in SFM. As expected, cell maturation caused a downregulation of CCR1 that was observed for all the media. In contrast, CCR7 is upregulated in SFM cultured cells and slightly downregulated in cells matured in RPMI. Importantly, despite the observed decrease, RPMI mDCS maintained a higher CCR7 expression than any of the SFM conditions (**Figure S4**). Regarding CCR5 expression, it was preserved in X-VIVO 15 and DendriMACS mDCs, decreasing in RPMI mDCs and surprisingly increasing in AIM-V mDCs.

**Figure 5:**
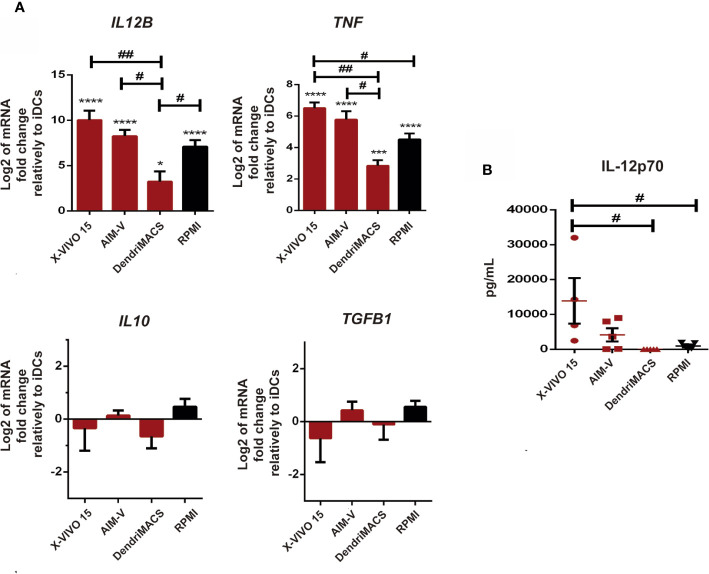
Impact of different culture media on mature dendritic cells (mDCs) transcription levels of *IL12B*, *TNF*, *IL10*, and *TGFB*, and on the production of IL12p70. Immature dendritic cells (iDCs) were maturated with a cocktail of TNF-α, IL-1β, Poly-I:C, and IFN-, for 24 h Then, both cells and culture supernatants were collected for analysis. **(A)** The transcription levels of *IL12B, TNF, IL10*, and *TGFB* were assessed by qPCR. Results are presented as mean log2 values of fold changes relatively to respective iDCs. Each value represents the mean ± SEM from four independent experiments. **(B)** The production of IL-12p70 by mDCs cultured in X-VIVO 15, AIM-V, DendriMACS, and RPMI was determined in culture supernatants by ELISA. Results are presented as mean ± SEM of at least four to five independent experiments. Statistical significance: *p < 0.05; ***p < 0.001; ****p < 0,0001 for iDCs vs mDCs cultured in the same medium; ^#^p < 0.05; ^##^p < 0.01 for comparison among mDCs.

Considering that cytokines produced by DCs are crucial to their immunomodulatory properties, we also characterize the impact of culture media on the transcription levels of *IL12B*, *TNF*, *IL10*, and *TGFB*, and on the release of IL-12p70 upon maturation. The transcription of anti-inflammatory *IL10* and *TGFB* was not significantly affected, while *IL12B* and *TNF* mRNA levels were strongly upregulated upon maturation in all tested media (**Figure 5A**). Among SFM, X-VIVO 15 and AIM-V supported the strongest increase in *IL12B* and *TNF* transcription, being significantly higher than those observed in DendriMACS mDCs. In accordance with these results, and despite some interindividual variability, DCs differentiated and matured in X-VIVO 15 or AIM-V were found to systematically produce higher amounts of IL-12p70 upon maturation (**Figure 5B**). Taken together, our data clearly indicate that from all the studied SFM, only X-VIVO 15 and AIM-V promoted an effective DC maturation with the used cocktail.

### Culture Media Influence the Functional Capacities of Produced DCs

**Figure 6:**
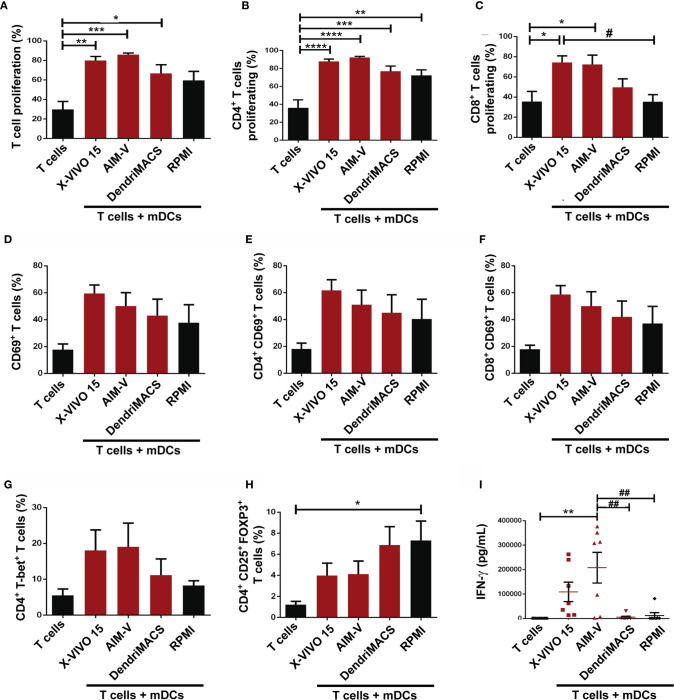
Functional capacities of mDCs generated in X-VIVO 15, AIM-V, DendriMACS and RPMI. **(A–C)** iDCs were treated for 24 h with maturation cocktail and then co-cultured with autologous T cells in a 1:10 ratio. Proliferation of T cells was determined after 5 days of co-culture by analyzing the percentage of total **(A)**, CD4^+^ **(B)** and CD8^+^ **(C)** T cells that presented a decrease in CFSE fluorescence (n=4). **(D–F)** Activation of total **(D)**, CD4^+^ **(E)**, and CD8^+^ **(F)** T cells was assessed by the expression of CD69 activation marker after 18 h of co-culture with mDCs. **(G, H)** The capacity of mDCs to polarize T cells toward Th1 (CD4^+^T-bet^+^) or Treg (CD4^+^CD25^+^FoxP3^+^) phenotype was assessed by flow cytometry. Results are expressed as percentage of Th1 **(G)** and Treg **(H)** cells within T lymphocytes. **(I)** The production of IFN-γ was determined by ELISA test in supernatants of 5 days co-cultures. Results are presented as mean ± SEM of at least four independent experiments. Statistical significance: *p < 0.05, **p < 0.01, ***p < 0.001, ****p < 0.0001 for T cells vs T cells + mDCs from each medium; ^#^p < 0.05; ^##^p < 0.01 for comparison among T cells + mDCs.

We also sought to address whether the capacities of DC to stimulate T cells were affected by the media used during their production. For this, allogenic mixed leucocyte reactions (MLR) were performed and T cell proliferation, phenotype and IFN-γ production were analyzed. As expected, independently of the used media, T cells that were stimulated with mDCs proliferated more than the T cells without stimulus (alone) (**Figures 6A–C**). Despite not being statistically significant, mDCs cultured in X-VIVO 15 and AIM-V induced a higher proliferation of T cells (79.98%±4.11; 85.93%±1.62, respectively) than mDCs cultured in DendriMACS and RPMI (66.48%±8.81; 59.25%±9.19, respectively). Looking at each lymphocyte population individually, mDCs cultured in AIM-V induced higher CD4^+^ T cell proliferation, while mDCs cultured in X-VIVO 15 induced superior proliferation of CD8^+^ T cells (**Figures 6B, C** **)**. Lymphoid activation antigen CD69 levels were also assessed to infer about T cells activation status (24). Culture media effects were similar to those observed for T cell proliferation with T cells (CD4^+^ and CD8^+^) stimulated with mDCs produced in X-VIVO 15 and AIM-V, presenting higher expression of CD69 (**Figures 6D–F**). The capacity of mDCs to polarize T cells toward Th1 or Treg was evaluated by the presence of T-bet^+^ and Foxp3^+^CD25^+^ cells, respectively, within the CD4^+^ T cell population. As shown in **Figures 6G**, **H**, mDCs cultured in X-VIVO 15 and AIM-V, elicited higher percentages of Th1 cells and a lower polarization of Treg, when compared to mDCs cultured in DendriMACS or RPMI. Accordingly, T cells stimulated with X-VIVO 15 and AIM-V mDCs were also found to produce significantly higher amounts of IFN-γ, a cytokine canonically produced by Th1 and cytotoxic T-lymphocytes (CTL) (**Figure 6I**). This superior capacity of X-VIVO 15 and AIM-V DCs to activate T cells strongly correlates with their more pronounced maturation status.

**Figure 7:**
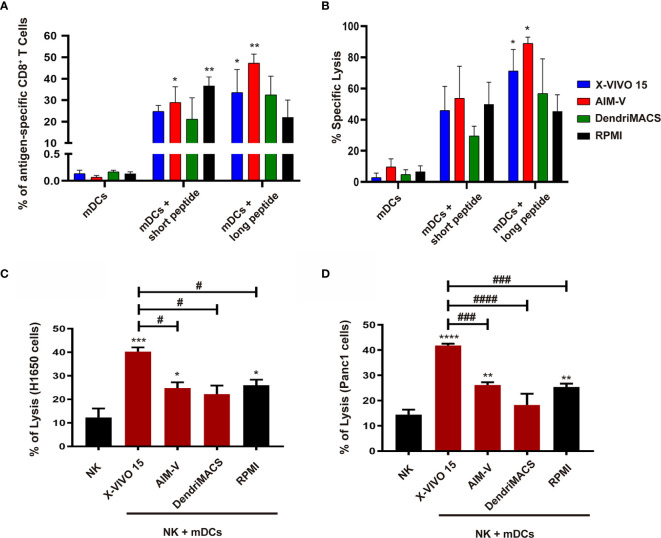
Priming of antigen-specific CD8^+^T cells and NK cytolytic activity after interaction with mDCs generated in X-VIVO 15, AIM-V, DendriMACS and RPMI. **(A, B)** iDCs were loaded with Melan-A short or long peptides and treated for 24 h with maturation cocktail and then co-cultured with autologous CD8^+^ and CD4^+^ cells for 14 days. **(A)** The percentage of primed and expanded Melan-A-specific CD8^+^ T cells was determined by flow cytometry using Pro5 HLA-A*02:01 – ELAGIGILTV Pentamer—Biotin plus Streptavidin-PE (n=3). **(B)** Primed CD8^+^ T cells were co-cultured with Melan-A-loaded H1650 lung cancer cell line at 50:1 ratio and their cytolytic activity was determined using LDH release assay (n=3). **(C, D)** iDCs were treated for 24 h with maturation cocktail and then co-cultured with autologous NK cells at a 1:5 ratio. After 24 h, NK cells were co-cultured with H1650 non-small cell lung cancer cell line **(C)** or Panc1 pancreatic cancer cell line **(D)** at 10:1 ratio, and their cytolytic activity was determined using LDH release assay (n=3). Results are presented as mean ± SEM. Statistical significance: *p < 0.05, **p < 0.01, ***p < 0.001, ****p < 0.0001 for mDCs vs mDCs + peptides or NK cells vs NK cells + mDCs from each medium; ^#^p < 0.05, ^###^p < 0.001, ^####^p < 0.0001 for comparison among different NK cells + mDCs conditions.

Next, we addressed the capacity of DCs to prime and expand antigen-specific CD8^+^ T cells followed by the analysis of their cytolytic activity over tumor cells. Co-culture of autologous T-cells with Melan-A loaded mDCs differentiated in X-VIVO 15 or AIM-V resulted in superior priming of antigen-specific CD8^+^ T cells (**Figure 7A**). Accordingly, these CD8^+^ T cells also shown enhanced cytolytic activity over cancer cells presenting Melan-A (**Figure 7B**). Finally, as DCs and NK cells establish a bidirectional crosstalk that enhances the activation status of both cell type, we analyzed DC maturation and NK activation after their interaction. The interaction with autologous NK cells resulted in enhanced maturation status of X-VIVO 15 and AIM-V mDCs (**Figure S5A**). In turn, mDCs produced in X-VIVO 15 were the more effectives in increasing NK activation markers CD69 and CD25 (**Figure S5B**). This correlates well with cytolytic assays since as shown in **Figures 7C, D**, NK cells co-cultured with X-VIVO 15 or AIM-V mDCs present higher cytolytic activity over Panc-1 and H1650 cancer cells.

### DCs Generated in Different Culture Media Have Distinct Metabolic Activity and Composition

**Figure 8:**
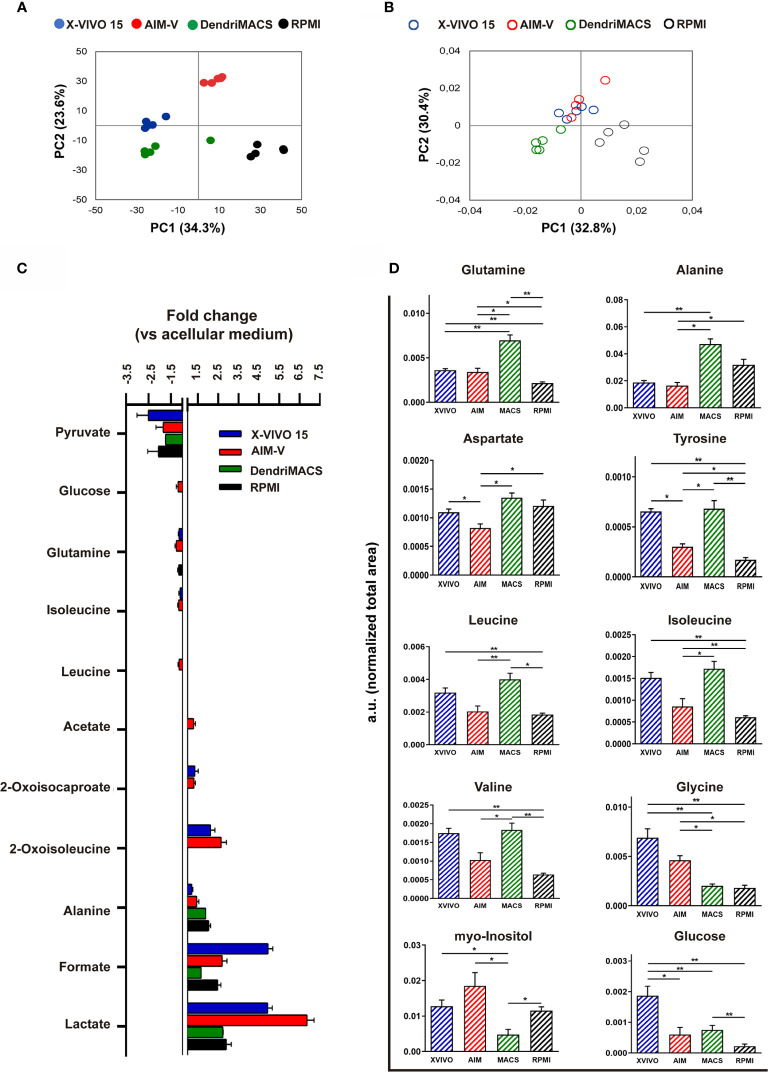
NMR-based metabolomic analysis of immature dendritic cells (iDCs) generated in different media. **(A)** Scores scatter plot obtained by principal component analysis (PCA) of spectral data from the different cell-conditioned media at day 6 of cell differentiation. **(B)** Scores scatter plot obtained by PCA of spectral data recorded for intracellular aqueous extracts of DCs cultured in different media for 6 days. **(C)** Metabolites consumed (negative bars) and excreted (positive bars) during the differentiation of Mo-DCs. Variations are expressed as fold change in relation to acellular media counterparts. **(D)** Relative intracellular levels of metabolites showing significant differences among the tested SFM, as assessed by integration of 1D ^1^H NMR spectra. Statistical significance: *p < 0.05, **p < 0.01 for comparison among DCs from the different media.

To assess the metabolic status of DCs produced in the different media, we performed an NMR metabolomic analysis of conditioned media (exometabolome) and of cell extracts (endometabolome), on day 6 of differentiation. The scores scatter plot resulting from applying principal component analysis (PCA) to the ^1^H‐NMR spectra of media supernatants evidenced a clear separation between groups, indicating substantial differences in media composition (**Figure 8A**). For a more detailed analysis of cells consumption/excretion behavior, spectral integration was carried out and metabolite variations were calculated by comparing each conditioned media with its acellular counterpart (**Figure 8C**). Pyruvate was the substrate preferred by all cells, being consumed to similar extent from all tested media (~2-fold decrease in cell-conditioned vs. acellular medium). Glutamine and isoleucine were only consumed by cells differentiated in X-VIVO 15 or AIM-V, while AIM-V DCs additionally consumed glucose and leucine. As for metabolites released into the medium, all SFM-differentiated cells excreted lactate, formate and alanine, whereas X-VIVO 15 and AIM-V DCs additionally released 2-oxoisocaproate and 2-oxoisoleucine (catabolic products of leucine and isoleucine). Acetate was excreted by AIM-V DCs only.

NMR analysis of cell extracts further showed DCs generated in different media to have distinct intracellular metabolic profiles. The PCA scores scatter plot (**Figure 8B**) shows that AIM-V and X-VIVO 15 DCs cluster together in the positive side of PC2, while DendriMACS and RPMI DCs, with scores in negative PC2, separate along the PC1 axis. The main quantitative differences between the 4 groups, as assessed by comparing normalized signal areas, are shown in **Figure 8D**. Most differences regarded the levels of amino acids. Among SFM-cultured cells, DendriMACS DCs displayed the highest levels of glutamine, alanine, aspartate, tyrosine, and branched chain amino acids, whereas AIM-V DCs contained the lowest amounts. On the other hand, intracellular levels of glycine were lower in DendriMACS DCs than in AIM-V and X-VIVO 15 DCs. Additionally, AIM-V and X-VIVO 15 DCs contained significantly higher levels of myo-inositol than DendriMACS DCs, while the glucose content was greater in X-VIVO 15 DCs than in all others. Intracellular levels of lactate, glutamate, formate and succinate did not vary significantly between SFM-cultured cells (**Figure S6**).

## Discussion

**Figure 9:**
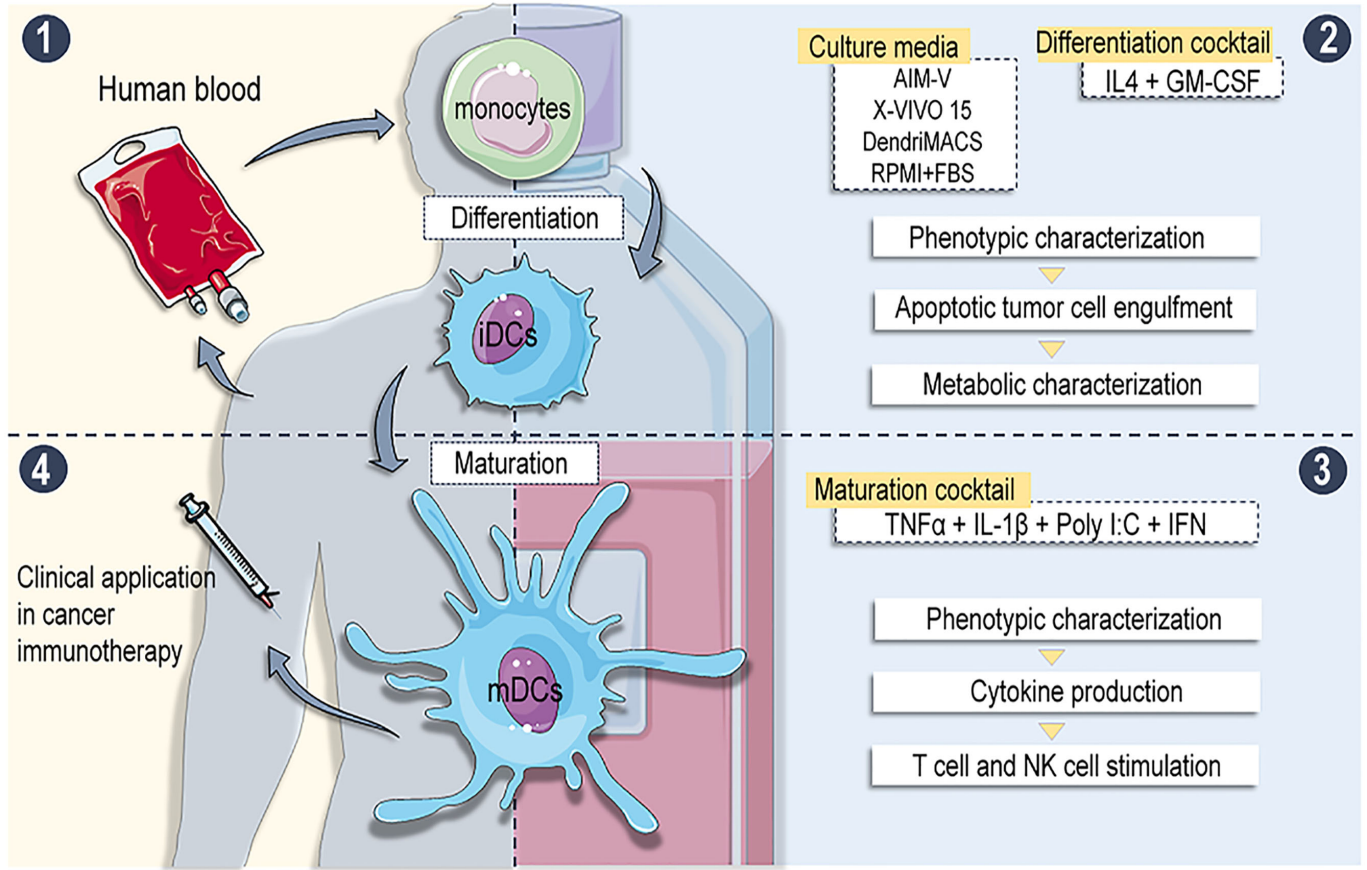
Overview of the work: (1) moDCs were differentiated from buffy coat isolated CD14+ monocytes using different serum free media (SFM) supplemented with IL-4 + GM-CSF. (2) Phenotype, apoptotic tumour engulfment capacity and metabolic profiles were analysed on immature DCs (iDCs). (3) iDCs were matured with alpha DC cocktail and phenotype, cytokine production and T and NK cells stimulatory abilities were assessed. (4) Results: Different SFM generate moDCs with distinct phenotypes and functional abilities. DCs with superior immunostimulatory abilities are produced in media evoking enhanced catabolism of amino acids and glucose. Conclusion: Definition of adequate culture medium is crucial for the production standardization of antitumor DC-based vaccines.

Fully defining culture conditions in which differentiation and maturation occur should be the first step toward the standardization of clinical grade DC production. In order to reduce the variability of a cellular product and to avoid possible immune reactions and transmission of infectious diseases, SFM are commonly used to produce clinical grade DCs (13, 14, 25). However, despite the likely impact of these SFM on the phenotype and functional abilities of produced DCs, very few studies directly compared them (13, 15). Here, we have shown that different SFM elicit a distinct metabolic state on DCs, conditioning cell phenotype, response to maturation stimuli and by consequence their T cell and NK cell immunostimulatory abilities (**Figure 9**). SFM were previously shown to support the differentiation of CD14^+^ monocytes into Mo-DCs without major impact in the cell yield and viability (12, 13, 15). In accordance with these observations, no differences were found between the yield obtained in the three tested SFM and FBS-supplemented RPMI. Nonetheless, the capacity of SFM-differentiated iDCs to engulf apoptotic/necrotic cells was clearly compromised. This can be due to the absence of serum bridging molecules, such as complement component C1q, Gas6, Protein S, and milk fat globule-EGF factor 8, that are known to indirectly mediate the recognition of dying cells by phagocytes (26–28). Thus, co-culture with necrotic tumor cells may not be the optimal antigen loading strategy for DCs produced in SFM.

Herein, we demonstrated that the phenotype of iDCs and mainly their behavior upon maturation presented substantial differences between tested media, which is then translated into distinct immunostimulatory capacities. When compared to DendriMACS DCs, cells produced in X-VIVO 15 or AIM-V were found to express higher levels of CD11c, CD86, and MHC-I, while presenting lower levels of CD1a and CD1c. Upon maturation, X-VIVO 15 and AIM-V DCs also expressed higher levels of CD86 and CCR7, presenting an enhanced capacity to secrete IL-12p70. In a previous work, DCs differentiated in serum-supplemented media were shown to present inferior levels of CD1a, when compared to cells differentiated in AIM-V (29). This difference was attributed to the activation of peroxisome proliferator-activated receptor γ (PPARγ) by components of lipoproteins present in serum or FBS. Our results do not corroborate these observations since all SFM elicited significantly lower levels of CD1a vs. FBS-supplemented RPMI. Therefore, lipids used in the formulation of SFM, namely mono- and polyunsaturated fatty acids, may be activating PPARγ in a way that exceeds the effect caused by serum lipoproteins (30). The expression of CD1a has also been positively correlated with a superior capacity of DCs to produce IL-12p70 and to promote Th1 polarization (31, 32). However, this assumption was recently challenged by data showing that genetic polymorphisms affect CD1a expression without compromising DCs Th1 polarizing capacities, or that IL-12p70 secretion and CD1a positivity are dissociated (33, 34). Our results support these latter observations, given that a higher expression of CD1a was found in RPMI and DendriMACS DCs. Nevertheless, these cells marginally produced IL-12p70 upon maturation and were clearly inferior in polarizing IFN-*γ*–producing T cells.

The low number of DCs present in T cell areas of lymphoid organs after intradermal or subcutaneous injection is pointed as a limiting factor to the efficacy of antitumor DC-based vaccines (35). Additionally, DCs expressing high levels of CCR7, the key regulator of DC migration toward secondary lymphoid tissues, elicit more effective antigen-specific immune responses and lower the required DC dosage during immunization (36, 37). Thus, DCs chemokine profile assumes particular relevance for the success of the clinical approach. In our experimental settings, CCR7 was slightly upregulated upon maturation in all tested SFM. CCR7 modulation on DCs is largely dependent on the stimuli used to mature cells. For instance, prostaglandin E2 (PGE2) was shown to be required for an effective receptor upregulation, with this molecule being part of the most frequently used maturation cocktail in clinical trials (IL1-β, IL-6, TNF-α; and PGE2) (38). However, PGE2 restrains the capacity of Mo-DCs to produce IL-12p70 (39). To surpass this drawback, the maturation cocktail used in this study has been proposed as a good compromise solution between migratory capacity and the production of the cytokine (40). Apart from the upregulation of CCR7, cell migration from peripheral tissues to lymph nodes also requires a coordinated downregulation of inflammatory chemokine receptors, such as CCR1 and CCR5. Accordingly, CCR1 was downregulated after DC maturation, independently of the media used. Surprisingly, DCs differentiated in AIM-V medium presented an increased expression of CCR5 upon maturation. Even though this may hamper the migration of injected DCs toward lymph nodes, it could facilitate their interaction with NK cells (41). This must be carefully contemplated, considering that in pre-clinical studies DC-NK crosstalk has been shown to be crucial for DC-based antitumor vaccines efficacy (42). Indeed, we showed that co-culture of AIM-V and X-VIVO 15 mDCs with autologous NK cells significantly enhanced their cytolytic activity. In accordance with the role played by IL-12p70 on NK cell activation (43), NK-activating capacity of AIM-V and X-VIVO 15 mDCs closely correlates with their higher production of this cytokine. Furthermore, as extensively reported in literature (44–47), we observed that DCs expressing higher levels of CD80, CD83 and CD86 (in our experimental settings those produced in AIM-V or X-VIVO 15) present superior capacity to polarize Th1 subset and to prime antigen-specific CD8^+^ T cells. Of note, DCs produced in all media tested showed the capacity to internalize and process long Melan-A peptide, cross-presenting resultant antigen on MHC-I molecules. This cross-presenting capacity is of particular relevance if tumor lysates or apoptotic tumor cells were used as antigen source during the production of the DC vaccine (5).

Recently, numerous evidence have demonstrated that cellular metabolism modulates DC functions, as well as their development and differentiation [reviewed in (8, 48)]. Differentiation of monocytes into Mo-DCs relies on the activation of mammalian target of rapamycin (mTOR) complex 1 (mTORC1) (49, 50), being supported by a catabolic metabolism dependent on oxidative phosphorylation (OXPHOS). In turn, upon activation, DCs shift to an aerobic glycolytic state where tricarboxylic acid (TCA) intermediates such as citrate, succinate and fumarate, contribute to the upregulation of co-stimulatory surface molecules, cytokine production and T cell stimulatory capacity (51, 52). In the present study, we have hypothesized that the phenotypical and functional differences observed between DCs produced in the different media could relate to their distinct metabolic status, induced during differentiation. One of the most noticeable differences, as assessed by metabolomics profiling, regarded amino acid metabolism. Unlike DendriMACS DCs, which did not use extracellular amino acids, AIM-V DCs and, to lower extent, X-VIVO 15 DCs were found to consume glutamine and branched chain amino acids (BCAA). Additionally, the intracellular levels of these and other amino acids were lower in X-VIVO 15 and/or AIM-V DCs, corroborating their higher catabolism. Glutamine consumption and intracellular use (glutaminolysis) likely reflect its anaplerotic channeling into the TCA cycle, to support OXPHOS and energy generation. As for BCAA, their catabolism has been recently reported to exacerbate the inflammatory properties of activated macrophages (53). Therefore, it is plausible to speculate that an increased catabolic rate of valine, leucine, and isoleucine in AIM-V or X-VIVO 15 DCs could contribute to their superior T cell stimulatory abilities. Another major difference between the DC groups compared regarded glucose consumption and intracellular levels. Only DCs differentiating in AIM-V consumed extracellular glucose, while showing the lowest intracellular levels, which, together with prominent lactate excretion, suggest higher glycolytic activity in these cells. This may possibly correlate with the observed higher basal expression of co-stimulatory molecules, as well as with the superior capacity of these cells to acquire a full mature phenotype following cytokine stimulation. Also, it should be noted that lactate, generated by LDH-catalyzed reduction of pyruvate, has been recently shown to be an important carbon source for CD8^+^ T cells proliferation and activation (54). As all cells took up pyruvate to similar extents, it is likely that higher lactate production and release originates from enhanced glutaminolysis, glycolysis and/or use of intracellular glycogen (55). Furthermore, AIM-V DCs were seen to excrete acetate, which could result from pyruvate, especially in conditions of metabolic overflow (56). Interestingly, acetate has been shown to be required for optimal CD8^+^ T cell function in the immune system (57). Finally, AIM-V and X-VIVO 15 DCs presented significantly higher intracellular levels of myo-inositol than DendriMACS DCs. This polyalcohol is a precursor molecule for inositol phosphates and other second messengers with important roles in T cell development (58), therefore, its putative relation to DCs immunostimulatory properties should not be ruled out.

## Conclusion

The present study delivers novel insights into the impact of culture conditions in the production of clinical grade DCs. Our data show that DC phenotype and functional abilities, such as the capacity to activate autologous NK cells and to prime antigen-specific CD8^+^ T cells, are strongly influenced by the GMP SFM used during DC differentiation and correlate with evoked metabolic status. Definition of culture conditions is therefore a critical step in the production of DCs for clinical application in cancer immunotherapy.

## Data Availability Statement

The data used and analysed in the current study are available from the corresponding author on reasonable request.

## Ethics Statement

Buffy coats were provided by the Portuguese Blood and Transplantation Institute (IPST) following an established protocol allowing access to buffy coats for scientific research with academic purposes only. The buffy coats were not specifically obtained for the present study and were provided without any personal detail from the donor.

## Author Contributions

MAC and BMN designed and conceived the study. JC, MAC, LM, CL, AT, and DF performed the experiments, acquired, and analyzed data. JC, MAC, LM, ID, and BMN interpreted data. JC and MAC wrote the original draft of the manuscript and BMN, CG, AF, and MTC made the final revisions and editing. All authors contributed to the article and approved the submitted version.

## Funding

This work was developed within the scope of iBiMED (UIDB/04501/2020) and CICECO-Aveiro Institute of Materials (UIDB/50011/2020 & UIDP/50011/2020), financed by national funds through the Foundation for Science and Technology (FCT). Funding was received from the project ImmunoDCs@CancerStemCells: Cellular Immunotherapy toward the elimination of cancer stem cells (Ref.: POCI-01-0247-FEDER-033532), co-funded by the European Regional Development Fund (FEDER), Competitiveness and Internationalization Operational Program (COMPETE2020) and Own Revenues of the University of Coimbra. João Calmeiro and Luís Mendes are supported by the FCT through individual PhD fellowships (PD/BDE/135076/2017; PD/BD/147220/2019). The NMR spectrometer is part of the National NMR Network (PTNMR), partially supported by Infrastructure Project N° 022161 (co-financed by FEDER through COMPETE 2020, POCI and PORL and FCT through PIDDAC).

## Conflict of Interest

JS and MC were employed by Tecnimede Group.

The authors declare that the research was conducted in the absence of any commercial or financial relationships that could be construed as a potential conflict of interest.

## Abbreviations

BCAA, brain chain amino acids; CCL19, chemokine ligand 19; CCL21, chemokine ligand 21; CCR, C-C chemokine receptor; CFSE, carboxyfluorescein succinimidyl ester; CSOP, clinical standard operating procedures; CTL, cytotoxic T-lymphocyte; CXCR4, C-X-C chemokine receptor type 4; DC, dendritic cell; ES, effect size; FBS, fetal bovine serum; FOXP3, forkhead-box-P3; GAPDH, glyceraldehyde 3-phosphate dehydrogenase; GM-CSF, granulocyte-macrophage colony-stimulating factor; GMP, good manufacturing practice; HIF-1α, hypoxia-inducible factor-1α; HLA, human leucocyte antigen; HMDB, human metabolome database; HS, human serum; iDC, immature dendritic cell; IFN-γ, interferon γ; IL, interleukin; IPST, Portuguese Blood and Transplantation Institute; LDH, lactate dehydrogenase; mDC, mature dendritic cell; MFI, mean fluorescence intensity; MHC, major histocompatibility complex; MLR, mixed leucocyte reaction; Mo-DC, monocyte-derived dendritic cell; mTOR, mammalian target of rapamycin; mTORC1, mammalian target of rapamycin complex 1; NK, natural killer; NMR, nuclear magnetic resonance; OXPHOS, oxidative phosphorylation; PBMCs, peripheral blood mononuclear cells; PBS, phosphate-buffered saline PCA, principal component analysis; PGE2, prostaglandin E2; Poly-I:C, polyinosinic:polycytidylic acid; PPARγ, peroxisome proliferator-activated receptor γ; qPCR, quantitative real-time polymerase chain reaction; RPMI, Roswell Park Memorial Institute Medium; SEM, standard error of the mean; SFM, serum-free media; T-bet, T-box protein expressed in T-cells; TCA, tricarboxylic acid; T cell, T-lymphocyte cell; TGFB1, transforming growth factor beta 1; Th1, T helper cell type 1; TNF, tumor necrosis factor; Treg, regulatory T cells.
