# Supplementary material for: In-Depth Analysis of the Impact of Different Serum-Free Media on the Production of Clinical Grade Dendritic Cells for Cancer Immunotherapy
Source: Front Immunol. 2021 Feb 5;11:593363. doi: 10.3389/fimmu.2020.593363 (PMC7893095; doi:10.3389/fimmu.2020.593363)
Supplement: Supplementary file 1 [file DataSheet_1.docx]

**Supplementary Material**

**Supplementary Table 1:** Primer sequences for studied genes

| **Gene Symbol** | **RefSeq ID** | **5’-3’ sequence**  **F: forward; R: reverse** |
| --- | --- | --- |
| *GAPDH* | NM_002046 | F: ACAGTCAGCCGCATCTTC  R: GCCCAATACGACCAAATCC |
| *IL12B* | NM_002187.2 | F: AAGTAGTTATGGCTAAGG  R: ATTGTGTATCAGGTTCAT |
| *TNF* | NM_000594 | F: CCTGTGAGGAGGACGAAC  R: CGAAGTGGTGGTCTTGTTG |
| *IL10* | NM_000572.2 | F: CATCTTCATCAACTACATAG  R: TGAGGTATCAGAGGTAAT |
| *TGFB1* | NM_000660.6 | F: GGAAACCCACAACGAAATC  R: GCTCTGATGTGTTGAAGAAC |


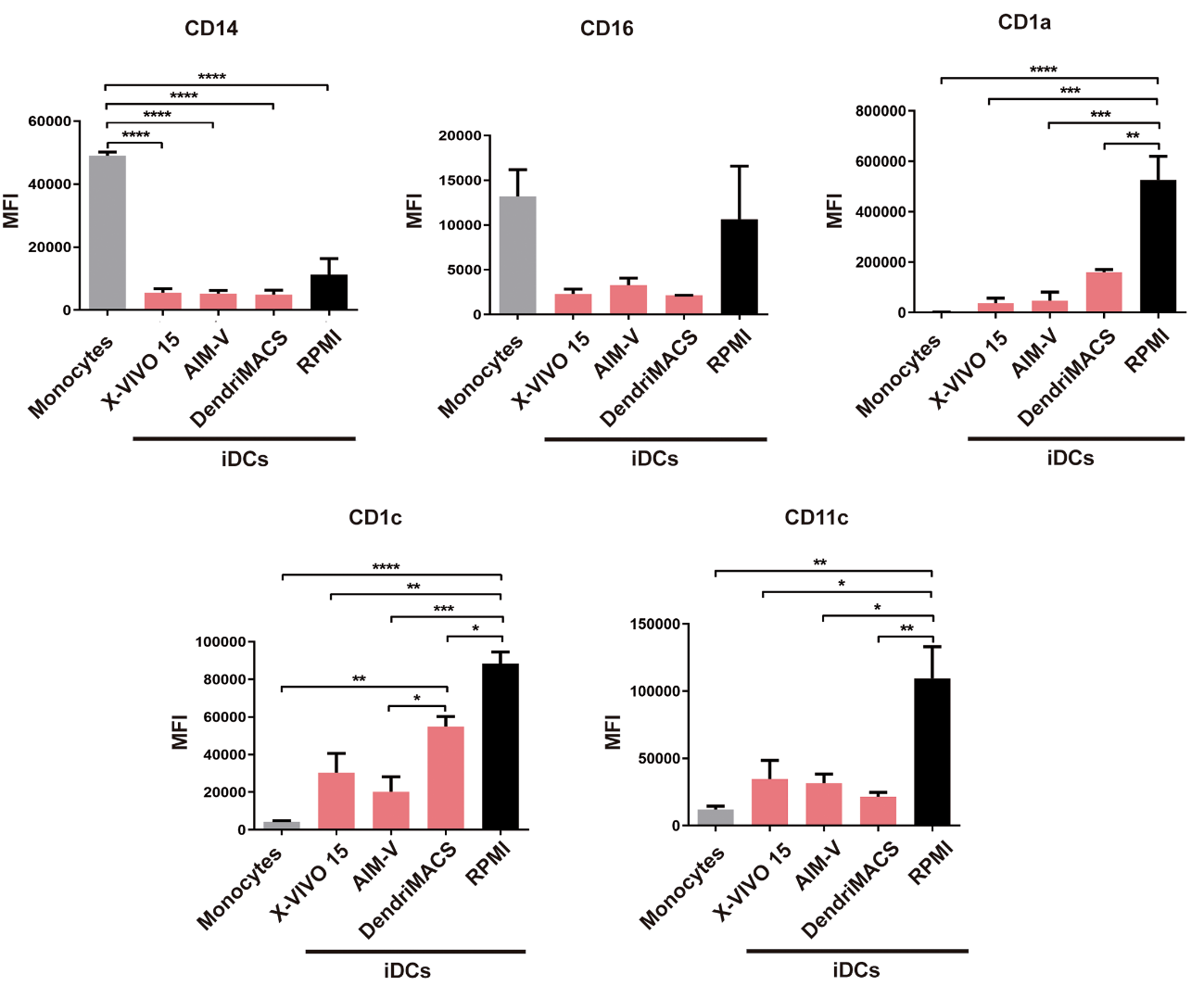


**Figure S1 – Mean fluorescence intensity (MFI) values of monocytes and iDCs cultured in 4 different media.** CD14, CD16, CD1c, CD1a and CD11c phenotypic differentiation markers were assessed by flow cytometry, with specific antibodies against selected markers. Results are presented as mean + SEM of at least three independent experiments. Statistical significance: *p < 0.05; **p < 0.01; ***p < 0.001; ****p < 0.0001


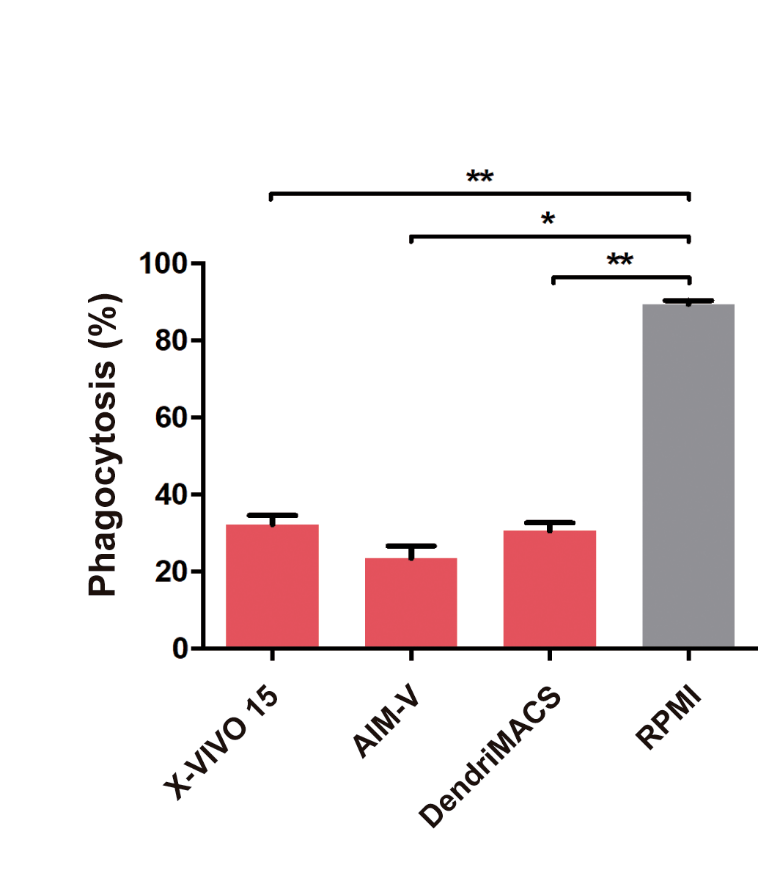


**Figure S2 – Effect of culture media on immature dendritic cells (iDCs) capacity to internalize apoptotic/necrotic tumour cells.** Monocytes were cultured with GM-CSF and IL-4 in three different good manufacturing practice (GMP) serum-free media (SFM) -AIM-V, X-VIVO-15 and DendriMACS - and with FBS-supplemented RPMI. After 6 days of culture, iDCs were compared regarding their capacity to internalize apoptotic/necrotic UM-UC-3 bladder carcinoma cells, as described in the *methods* section. Endocytosis percentage was analysed by flow cytometry and was estimated based on the positivity for CFSE staining within MHC-II^+^ DCs. Results are presented as mean ± SEM of at least three independent experiments. Statistical significance: *p<0,05; **p<0,01.


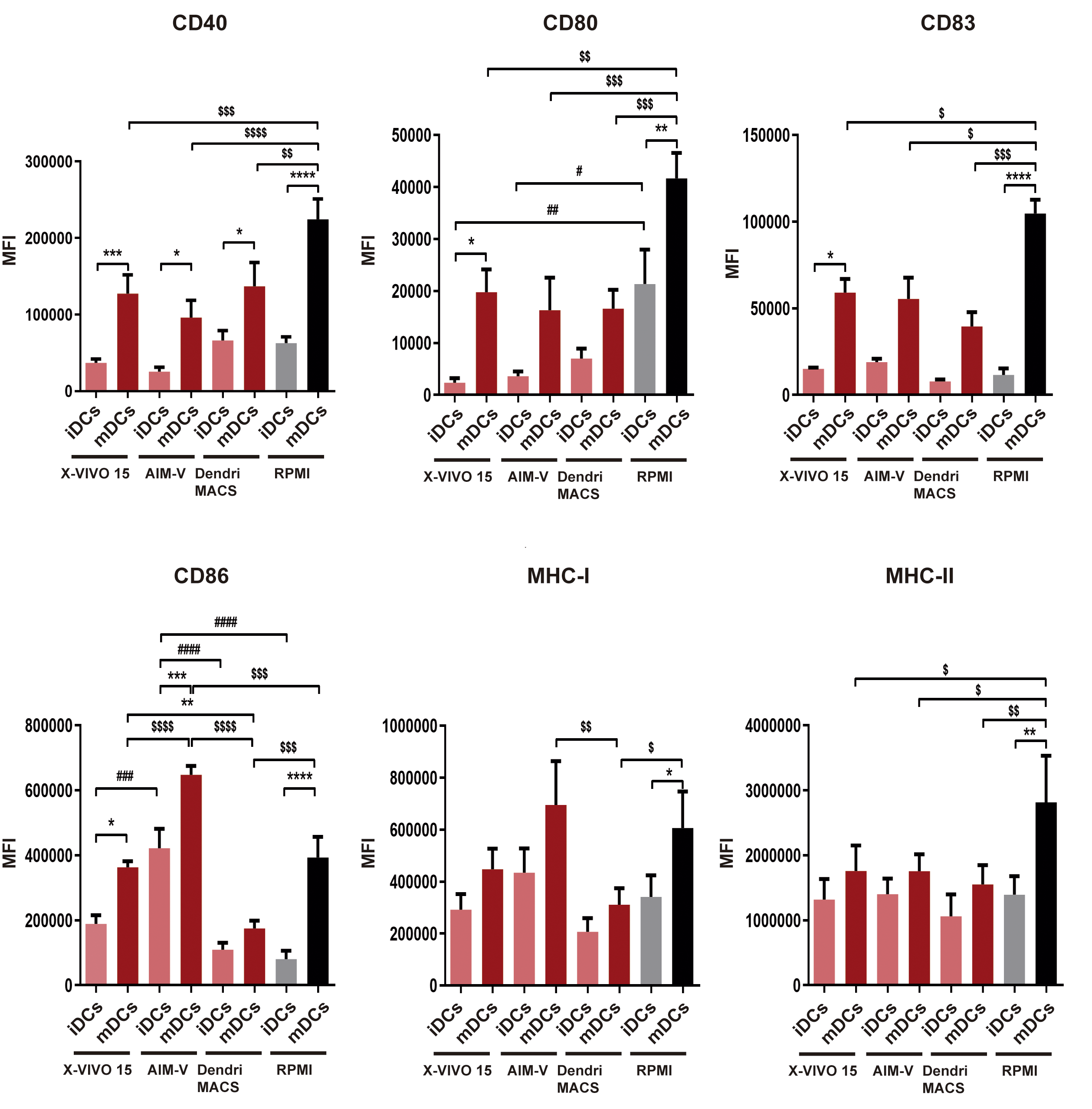


**Figure S3 – Mean fluorescence intensity (MFI) values of iDCs and mDCs cultured in 4 different media.** CD80, CD83, CD86, CD40, MHC-I and MHC-II phenotypic maturation markers were assessed by flow cytometry. Results are presented as mean + SEM of at least four independent experiments. Statistical significance: *p < 0.05; **p < 0.01; ***p < 0.001; ****p < 0.0001 iDCs vs mDCs from the same media; #p < 0.05; ##p < 0.01; ###p < 0.001; ####p < 0.0001 iDCs vs iDCs among different media; §p < 0.05; §§p < 0.01; §§§p < 0.001; §§§§p < 0.0001 mDCs vs mDCs among different media.


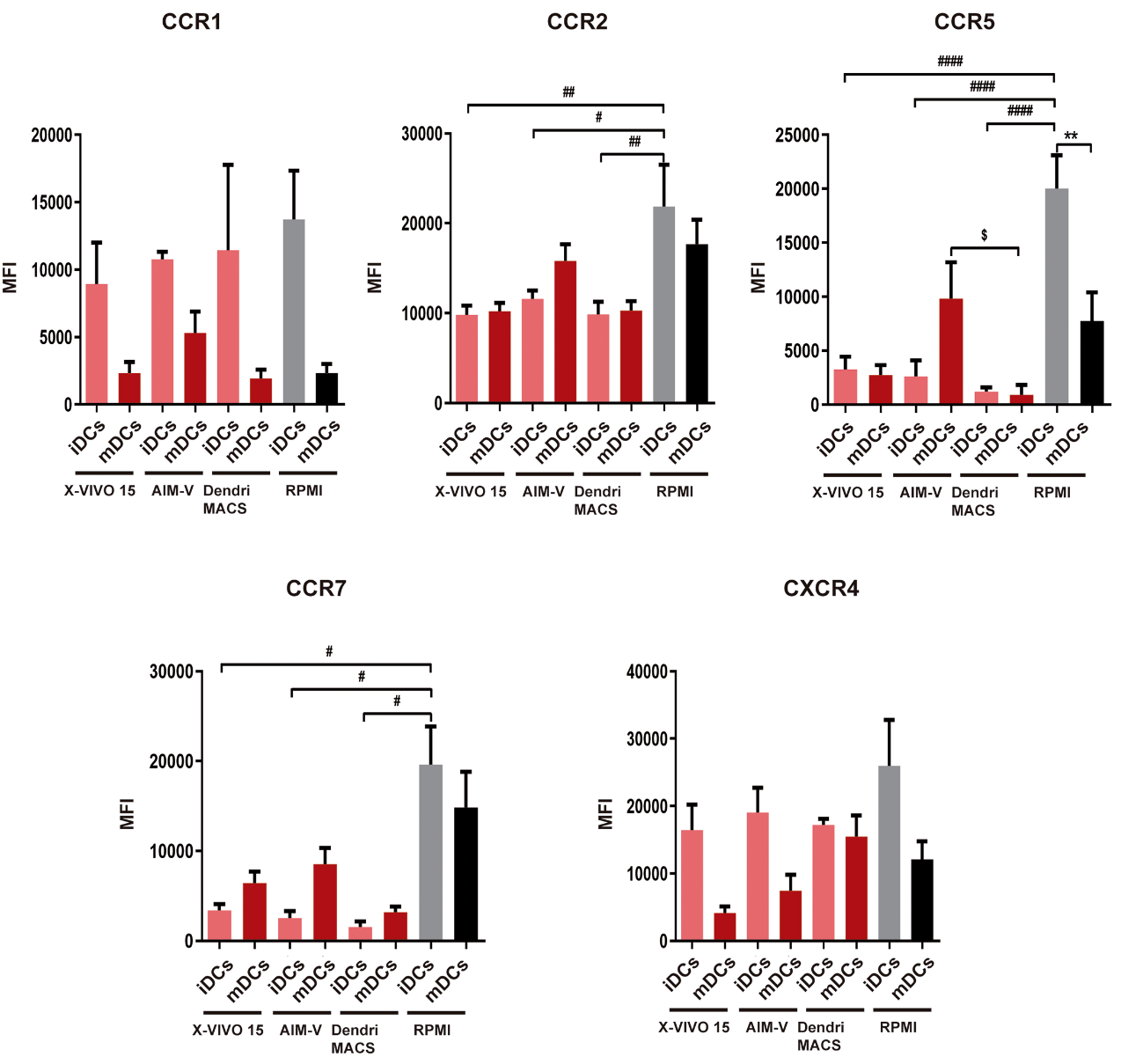


**Figure S4 – Mean fluorescence intensity (MFI) values of iDCs and mDCs cultured in 4 different media.** CCR1, CCR2, CCR5, CCR7 and CXCR4 surface expression levels were assessed by flow cytometry. Results are presented as mean + SEM of at least four independent experiments. Statistical significance: *p < 0.05 iDCs vs mDCs for the same media; #p < 0.05; ##p < 0.01; ###p < 0.001; ####p < 0.0001 iDCs vs iDCs among different media; §p < 0.05 mDCs vs mDCs among different media;


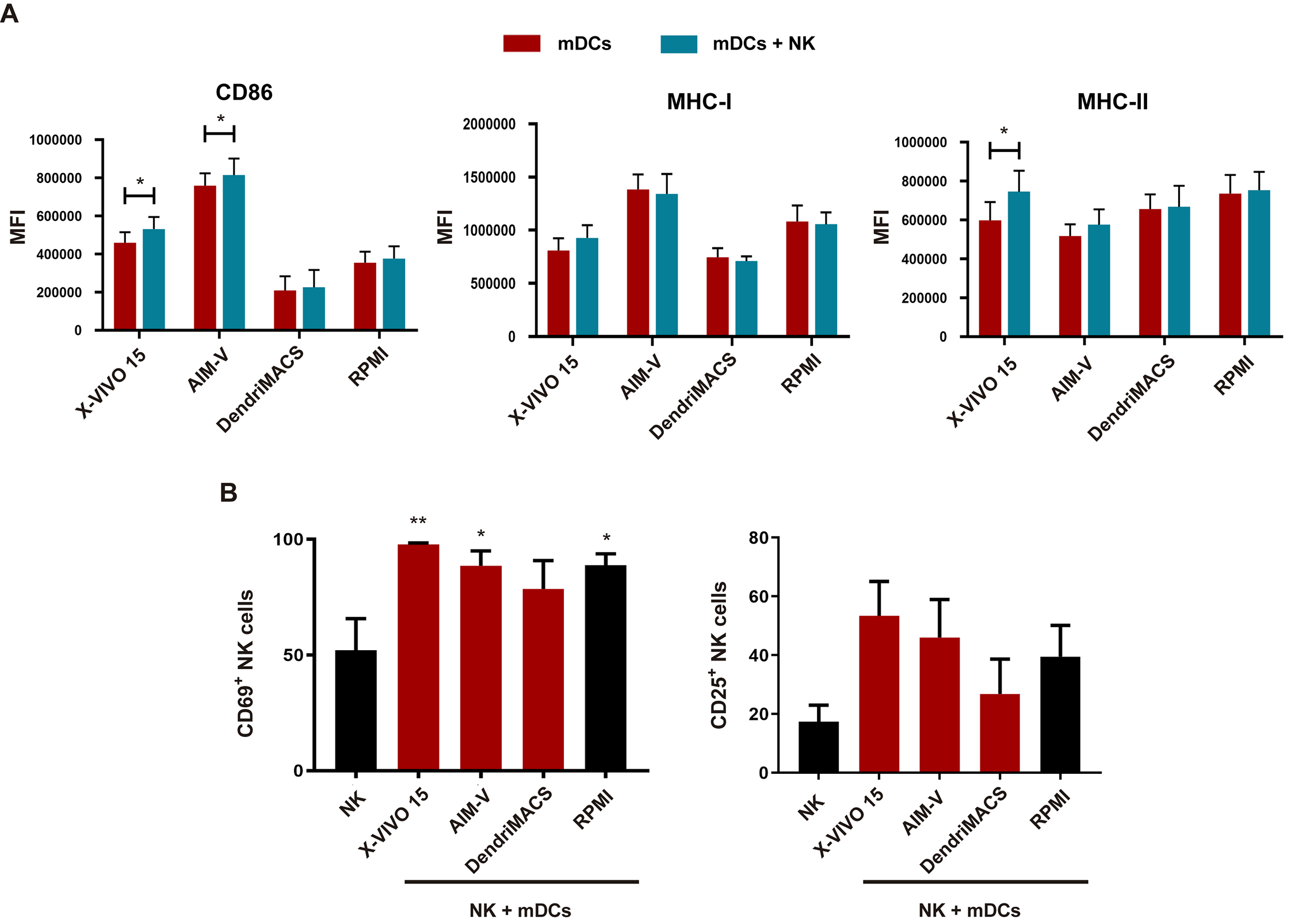


**Figure S5 – Effect of DC-NK crosstalk on activation status.** CD86, MHC-I and MHC-II phenotypic DC maturation markers (A), as well as CD25 and CD69 NK activation markers (B) were assessed by flow cytometry after DC-NK cell co-culture. Results are presented as mean + SEM of at least three independent experiments. Statistical significance: *p < 0.05; **p < 0.01; ***p < 0.001; ****p < 0.0001


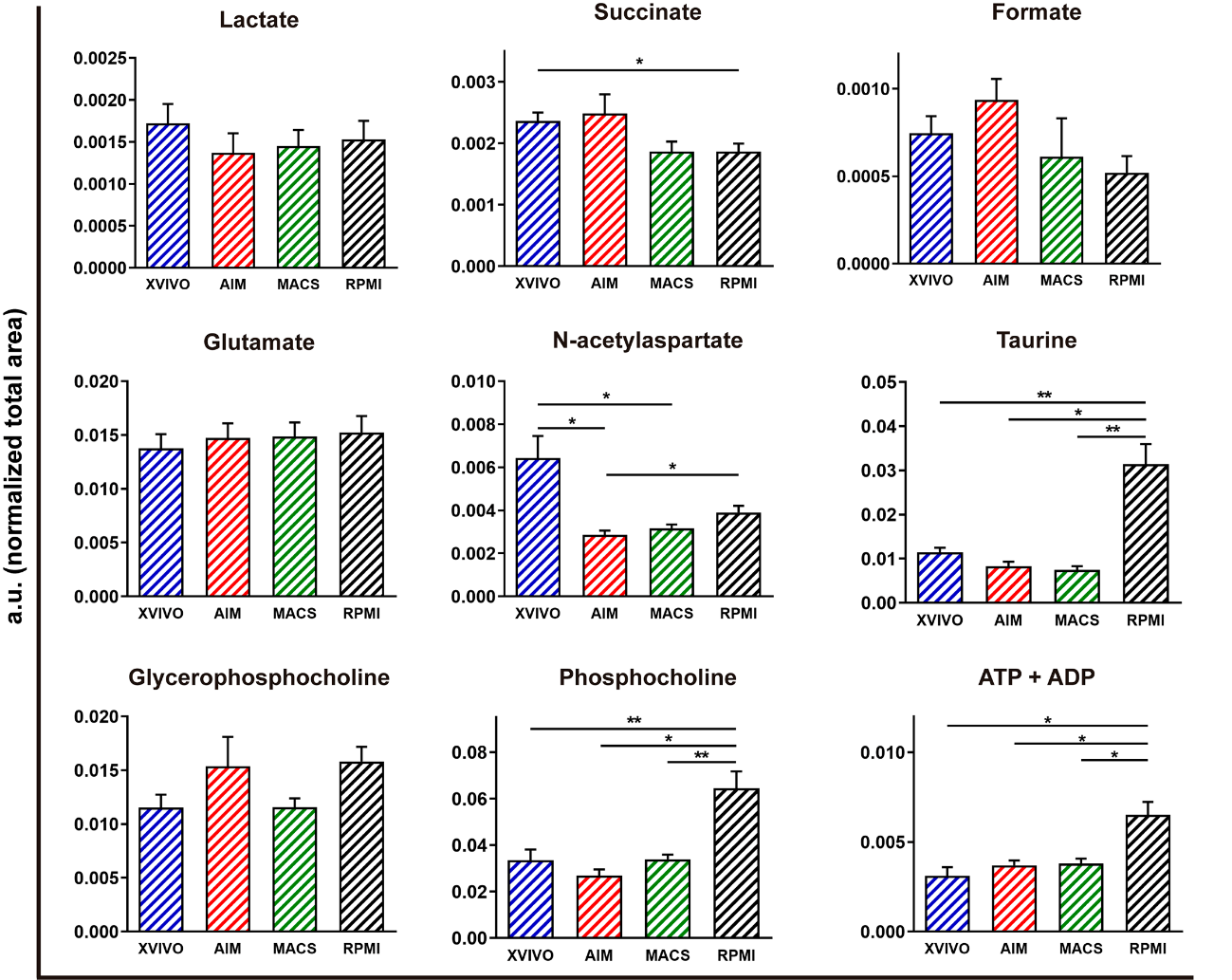


**Figure S6 - NMR-based metabolomic analysis of immature dendritic cells (iDCs) generated in different media.** Relative intracellular levels of metabolites (complementary to Figure 8) among the tested SFM, as assessed by integration of 1D ^1^H NMR spectra. Statistical significance: *p < 0.05; **p < 0.01
